# MeCP2 inhibits cell functionality through FoxO3a and autophagy in endothelial progenitor cells

**DOI:** 10.18632/aging.102183

**Published:** 2019-09-02

**Authors:** Siyuan Zha, Zhen Li, Shuyan Chen, Fang Liu, Fei Wang

**Affiliations:** 1 Department of Geriatrics, Xinhua Hospital, School of Medicine, Shanghai Jiao Tong University, Shanghai, China

**Keywords:** EPCs, autophagy, MeCP2, FoxO3a, epigenetics

## Abstract

Objectives: Autophagy is an evolutionarily conserved intracellular degradation mechanism in which cell constituents are phagocytosed to maintain cellular homeostasis. Forkhead box O 3a (FoxO3a) promotes autophagy to protect cells from environmental stress. Methylated CpG binding protein 2 (MeCP2) is a nuclear protein that binds DNA and represses transcription. However, the mechanism and interplay between FoxO3a and MeCP2 underlying endothelial progenitor cell (EPC) function are not fully understood.

Results: In EPCs, MeCP2 overexpression attenuated autophagy and cell functionality, which were reversed by the autophagy activator rapamycin or co-transfection with FoxO3a. FoxO3a promoted cell function, which was reversed by the autophagy inhibitor chloroquine. Following MeCP2 overexpression, MeCP2 was found enriched on the FoxO3a promoter, resulting in promoter hypermethylation and enhanced H3K9 histone modification in nucleosomes of the FoxO3a promoter.

Conclusions: MeCP2 attenuated cell functionality via DNA hypermethylation and histone modification of the FoxO3a promoter to inhibit FoxO3a transcription and autophagy.

Materials and Methods: EPCs were isolated from human umbilical cord blood and treated with adenoviral vectors containing interference sequences. The effects and mechanism of MeCP2 and FoxO3a were analyzed by utilizing western blotting, cell counting kit-8, transwell plates, Matrigel, matrix adhesion, transmission electron microscopy, and chromatin immunoprecipitation.

## INTRODUCTION

Autophagy is an evolutionarily conserved intracellular degradation process where cell constituents are enclosed within double-membraned vesicles and transported to lysosomes [1]. Recent research has suggested that autophagy is intimately associated with cellular homeostasis and a decrease in cellular functionality during senescence [2–4]. Thus, elucidating autophagic mechanisms and regulating cellular function are of great significance [5, 6]. LC3 II is closely correlated with the extent of autophagosome formation, indicating that LC3 II may be a marker of autophagic activity [7]. Moreover, Beclin1 is known to play an important role in the recruitment of other autophagic proteins during expansion of the pre-autophagosomal membrane and structure [8], while p62 is a bona fide autophagy substrate that accumulates during autophagy inhibition [9, 10]. Therefore, LC3 II, Beclin1, and p62 were used to evaluate autophagy levels. In addition, autophagy-related 5 (ATG5) and autophagy-related 7 (ATG7) were used to supplement estimates of autophagy levels in our studies [11].

In vertebrates, CpG DNA methylation is very important as it is involved in fine-tuning gene expression, silencing of retrotransposable elements, and genomic stability [12, 13]. Methylated CpG binding protein 2 (MeCP2) is a nuclear protein—named for its ability to selectively recognize and bind to DNA—that is particularly abundant in neuronal cells. At first, research on MeCP2 focused on the nervous system due to its high expression levels in neuronal cells. In recent years however, research on MeCP2 has expanded beyond the nervous system to also include digestive, endocrine, and cardiovascular systems [14–18]. MeCP2 was initially proposed to mediate transcriptional repression by binding to the co-repressor Sin3A and recruiting histone deacetylase (HDAC) to methylated promoters, creating a hypoacetylated locally repressive chromatin environment [19–22]. In addition, through histone modifications modulating the degree of nucleosome compaction, MeCP2 can affect chromatin accessibility to various factors, particularly transcriptional regulators [23, 24]. Furthermore, it has been demonstrated that histone 3 lysine 9 dimethylation (H3K9me2) is associated with transcription repression [25].

Forkhead box O 3a (FoxO3a) is an important transcriptional regulator involved in various cellular functions. Previous studies have shown that FoxO3a is a tumor suppressor that regulates the expression of genes involved in apoptosis, cell cycle arrest, and oxidative stress resistance. In general, FoxO3a suppresses cell cycle progression and promotes cell death [26, 27]. However, recent studies have discovered that FoxO3a is associated with longevity and autophagy. Interestingly, FoxO3a increases autophagy to protect cells from environmental stress [28–30]. Thus, FoxO3a may potentially have a protective role in maintaining cell homeostasis, which is unlike its established role [31, 32].

There is little research on the crosstalk between MeCP2 and FoxO3a in EPCs, which repair injured endothelial cells and play an important role in atherosclerosis [33]. Therefore, we analyzed the changes in MeCP2 and FoxO3a expression levels during replicative senescence in EPCs and further examined the effect of MeCP2 and FoxO3a on autophagy and cell functionality.

## RESULTS

### EPC identification

**Figure 1:**
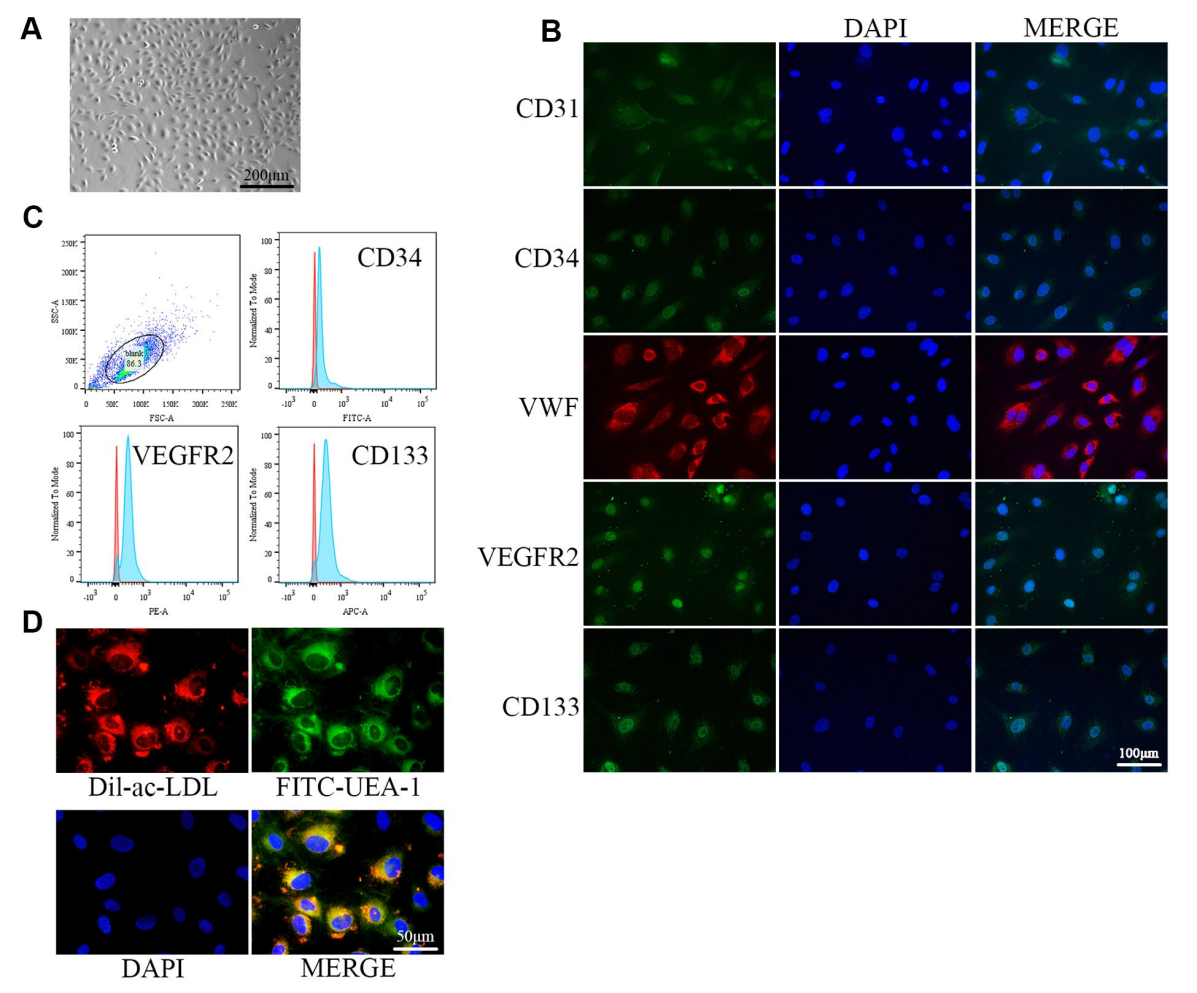
**Identification of EPCs from human umbilical cord blood.** (**A**) EPCs at passage 1 (P1) exhibited monolayer growth and cobblestone morphology after 2 weeks. (**B**) Cells were characterized by the immunofluorescence detection of CD31, CD34, VWF, VEGFR2, and CD133. (**C**) Flow cytometry revealed the positive expression rates of CD34, VEGFR2, and CD133. (**D**) Uptake of ac-LDL and binding of FITC-UEA-1 are characteristic functions of endothelial cells.

The isolated cells exhibited monolayer growth and cobblestone morphology, which is typical of EPCs (Figure 1A). Immunofluorescence confirmed the cells as EPCs based on expression of the cellular surface antigens CD31, CD34, VWF, VEGFR2, and CD133, which are standard EPC markers (Figure 1B) [34]. Flow cytometry analysis revealed that the positive expression rates of CD34, VEGFR2, and CD133 were 91.2%, 95.0%, and 95.3%, respectively (Figure 1C). VEGFR2 is an endothelial cell marker, while CD34 and CD133 are progenitor cell markers [25]. EPCs were further verified by examining Dil-Ac-LDL uptake and FITC-UEA-1 binding, which are characteristic functions of endothelial cells (Figure 1D) [35].

### Changes in protein expression during replicative senescence

**Figure 2:**
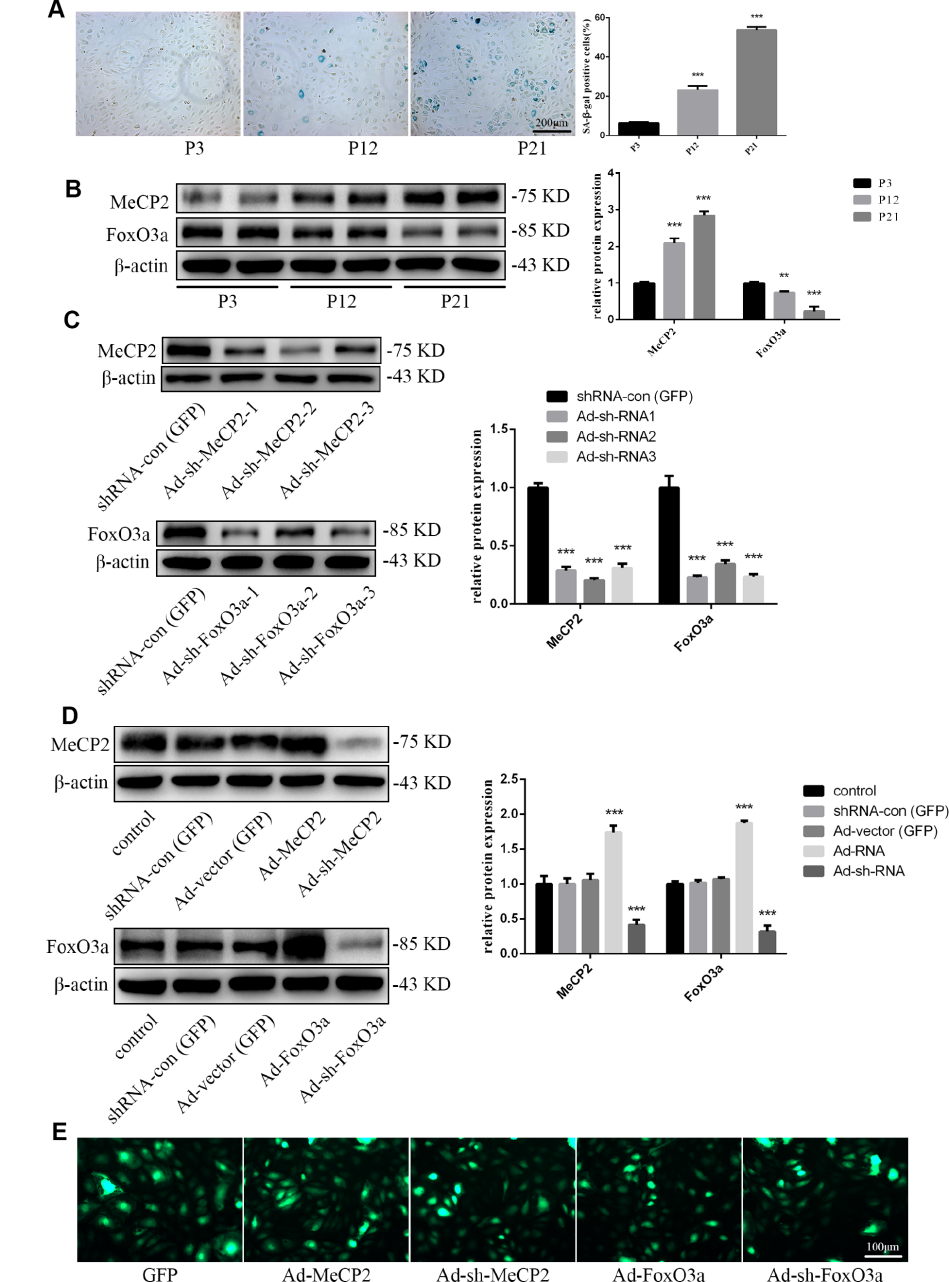
**Protein expression during replicative aging and the efficacy of adenoviral vectors containing interference sequences.** (**A**) Senescence-associated beta-galactosidase (SA-β-gal) staining during P3, P12, and P21 confirmed the increasing rate of aging EPCs during replicative senescence. (**B**) Protein levels of MeCP2 and FoxO3a were detected by western blotting during replicative senescence. (**C**) Protein levels of MeCP2 and FoxO3a were detected by western blotting after transfection with Ad-sh-MeCP2 or Ad-sh-FoxO3a. (**D**) Protein levels of MeCP2 and FoxO3a were detected by western blotting after overexpression and silencing of MeCP2 or FoxO3a. (**E**) Efficacy of adenoviral vectors was confirmed under fluorescence microscopy. *P < 0.05, **P < 0.01, and ***P < 0.001 vs. control.

For our senescence experiments, we repeatedly subcultured EPCs for up to 21 passages (P21). P1 cells were small and displayed typical cobblestone-like morphology. However, after repeated subculturing, P21 cells were larger and more irregular in shape, as some were branch-like and polygonal or long and spindle-shaped (Supplementary Figure 1A). Moreover, proliferation rates for EPCs was attenuated (Supplementary Figure 1B). Senescence was confirmed through morphology and SA-β-gal assays; SA-β-gal positivity increased with repeated subculture (Figure 2A). Western blot analysis for protein expression levels indicated that MeCP2 levels increased while FoxO3a levels decreased with repeated subculture (Figure 2B).

To verify the effects of MeCP2 and FoxO3a associated with replicative senescence, we employed adenoviral vectors harboring Ad-MeCP2, Ad-sh-MeCP2, Ad-FoxO3a, or Ad-sh-FoxO3a to transfect EPCs. Prior to that, we compared the effect of sh-RNA1, 2, and 3 (Figure 2C) and used the most efficient sh-RNA in subsequent experiments. GFP, Ad-MeCP2, Ad-sh-MeCP2, Ad-FoxO3a, and Ad-sh-FoxO3a were further used to confirm the efficacy of adenoviral vectors (Figure 2D, 2E).

### FoxO3a promotes autophagy and EPC functionality

**Figure 3:**
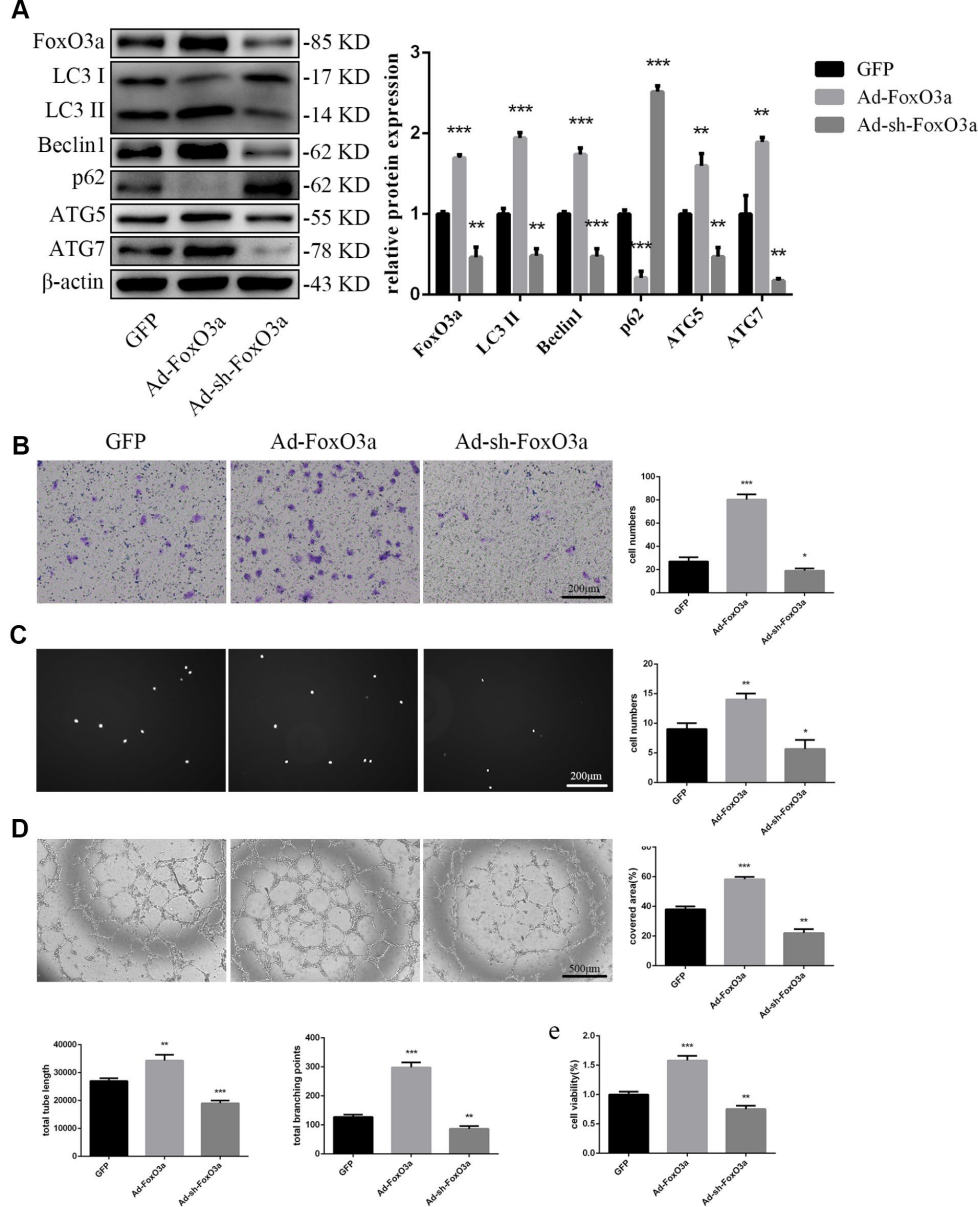
**FoxO3a promoted autophagy and cellular function.** (**A**) LC3 II, Beclin1, p62, ATG5, and ATG7 protein levels were detected by western blotting after transfection with Ad-FoxO3a or Ad-sh-FoxO3a for 48 h. (**B**) Cell migration was evaluated by Transwell migration assays after transfection with Ad-FoxO3a or Ad-sh-FoxO3a for 48 h. (**C**) Cell adhesion ability was evaluated by matrix adhesion assays after transfection with Ad-FoxO3a or Ad-sh-FoxO3a for 48 h. (**D**) Angiogenic ability was evaluated by Matrigel assays after transfection with Ad-FoxO3a or Ad-sh-FoxO3a for 48 h. (**E**) Cell viability was evaluated with CCK-8 after transfection with Ad-FoxO3a or Ad-sh-FoxO3a for 48 h. *P < 0.05, **P < 0.01, and ***P < 0.001 vs. control.

To examine the effects of FoxO3a, we used adenoviral vectors overexpressing FoxO3a and found that LC3 II, Beclin1, ATG5, and ATG7 levels were increased. However, FoxO3a overexpression attenuated p62 levels (Figure 3A). Our findings suggest that FoxO3a promoted autophagy. Moreover, FoxO3a overexpression also promoted cell migration, adherent ability, angiogenic ability, and cell viability (Figure 3B–3E). The opposite effects were observed in the group treated with Ad-sh-FoxO3a.

### MeCP2 attenuates autophagy and EPC functionality

**Figure 4:**
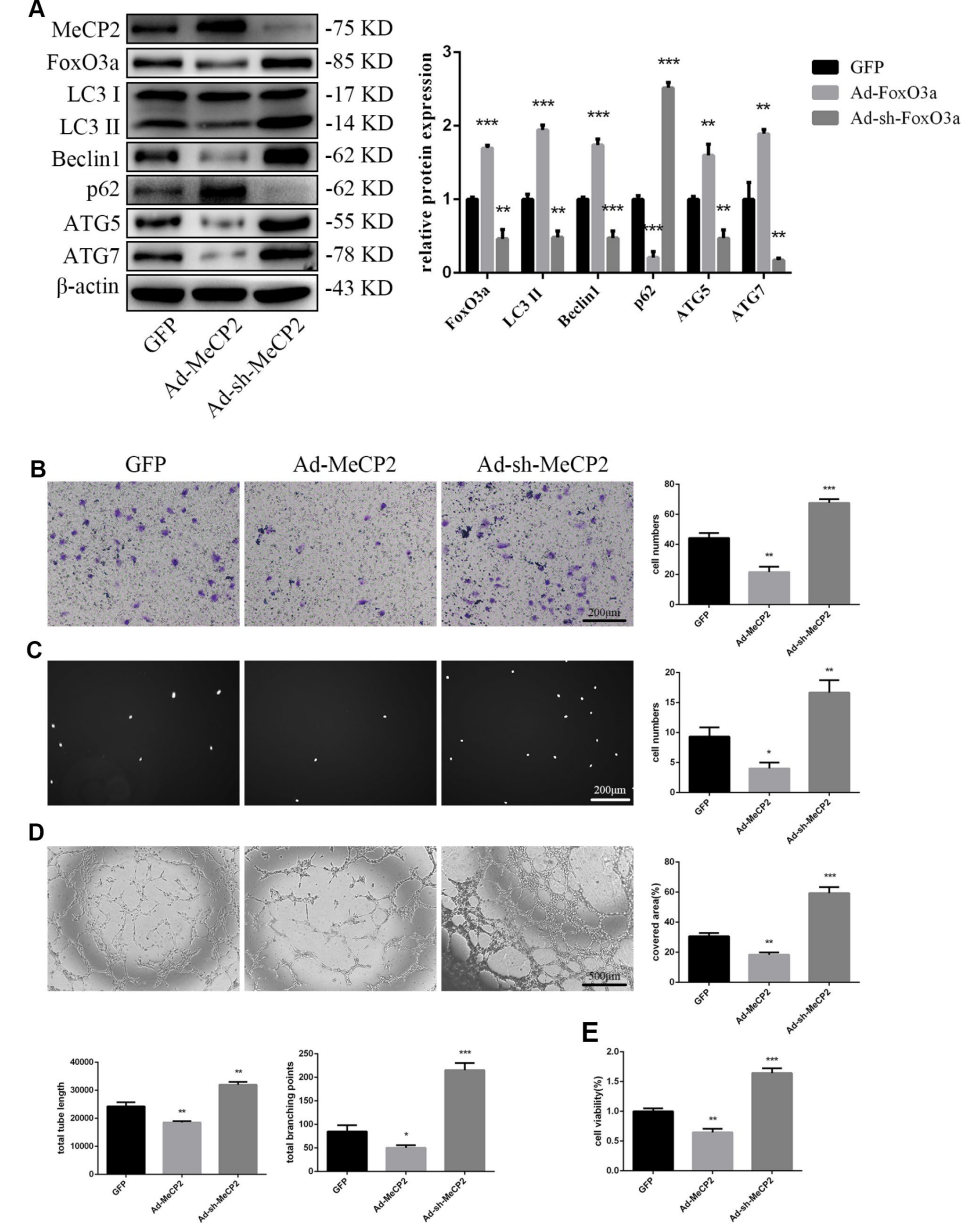
**MeCP2 inhibited FoxO3a, autophagy, and cellular function.** (**A**) Protein levels of FoxO3a, LC3 II, Beclin1, p62, ATG5, and ATG7 were detected by western blotting after transfection with Ad-MeCP2 or Ad-sh-MeCP2 for 48 h. (**B**) Cell migration was evaluated by Transwell migration assays after transfection with Ad-MeCP2 or Ad-sh-MeCP2 for 48 h. (**C**) Cell adhesion ability was evaluated by matrix adhesion assays after transfection with Ad-MeCP2 or Ad-sh-MeCP2 for 48 h. (**D**) Angiogenic ability was evaluated by Matrigel assays after transfection with Ad-MeCP2 or Ad-sh-MeCP2 for 48 h. (**E**) Cell viability was evaluated with CCK-8 after transfection with Ad-MeCP2 or Ad-sh-MeCP2 for 48 h. *P < 0.05, **P < 0.01, and ***P < 0.001 vs. control.

To examine the effects of MeCP2, we overexpressed MeCP2 using adenoviral vectors and found that FoxO3a, LC3 II, Beclin1, ATG5, and ATG7 levels decreased, while p62 levels were elevated (Figure 4A). Thus, these results suggest that MeCP2 inhibits autophagy. In addition, MeCP2 overexpression attenuated cell migration, adherent ability, angiogenic ability, and cell viability (Figure 4B–4E). The opposite effects were observed in the group treated with Ad-sh-MeCP2.

### FoxO3a promotes EPC functionality through autophagy

**Figure 5:**
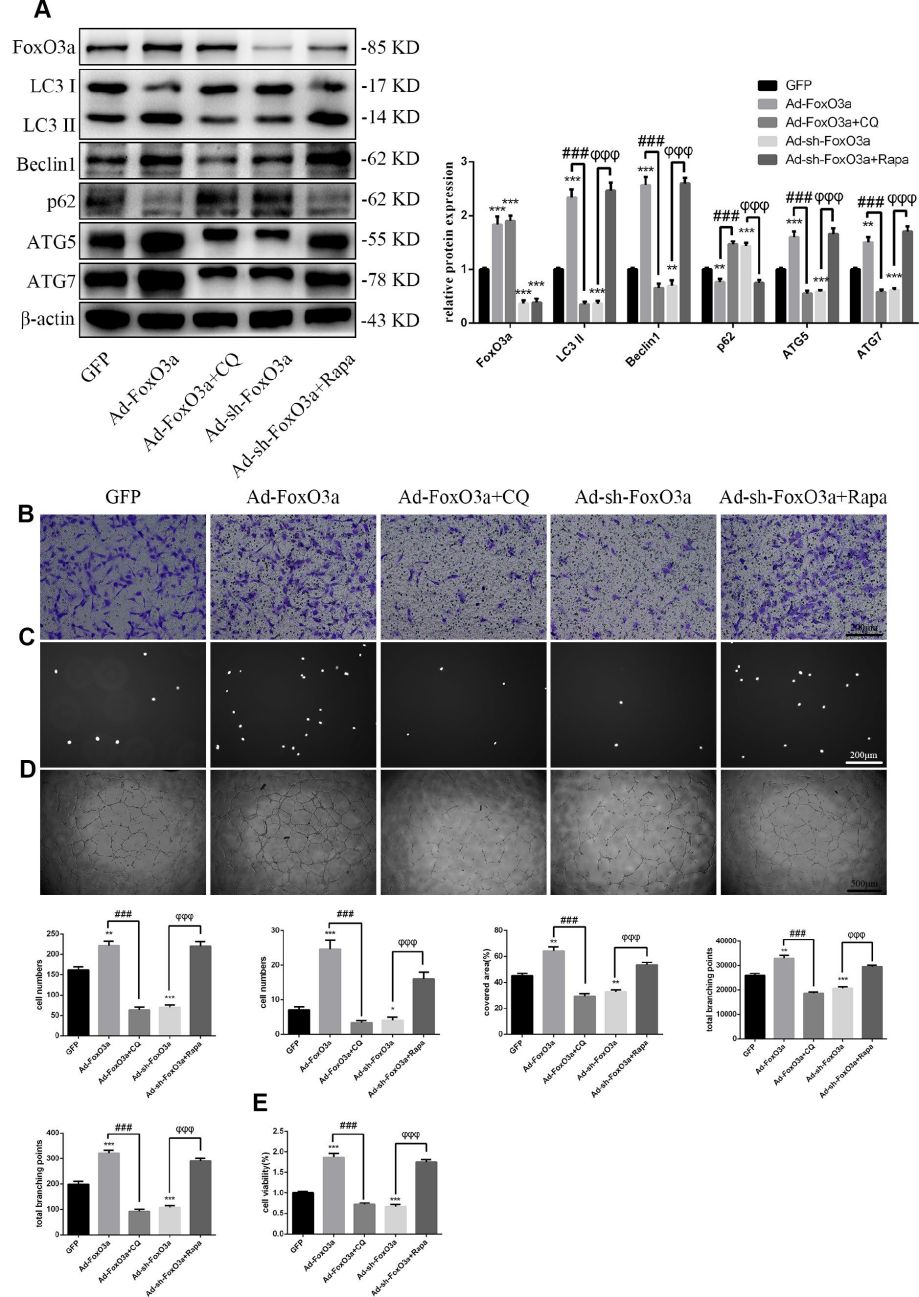
**FoxO3a promotes EPC functionality through autophagy.** (**A**) LC3 II, Beclin1, p62, ATG5, and ATG7 protein levels were detected by western blotting after GFP, Ad-FoxO3a, Ad-FoxO3a + CQ, Ad-sh-FoxO3a, or Ad-sh-FoxO3a + Rapa. (**B**) Cell migration was evaluated by Transwell migration assays after treatment for 48 h. (**C**) Cell adhesion ability was evaluated by matrix adhesion assays after treatment for 48 h. (**D**) Angiogenic ability was evaluated by Matrigel assays after treatment for 48 h. (**E**) Cell viability was evaluated with CCK-8 after treatment for 48 h. *P < 0.05, **P < 0.01, and ***P < 0.001 vs. control. ^#^P < 0.05, ^##^P < 0.01, and ^###^P < 0.001 vs. Ad-FoxO3a treatment. ^φ^P < 0.05, ^φφ^P < 0.01, and ^φφφ^P < 0.001 vs. Ad-sh-FoxO3a treatment.

Our results indicated that FoxO3a promotes autophagy and EPC functionality. Therefore, to analyze the effects of autophagy on EPC function, we used the autophagy activator Rapa or the autophagy inhibitor CQ to block the effects of FoxO3a. Our results revealed that autophagy promoted by Ad-FoxO3a was inhibited after CQ treatment and that autophagy attenuated by Ad-sh-FoxO3a increased after Rapa treatment (Figure 5A). Correspondingly, cell functionality results were found to be the opposite of the changes in autophagy observed in groups treated with CQ or Rapa (Figure 5B–5E).

### MeCP2 attenuates EPC functionality through autophagy

**Figure 6:**
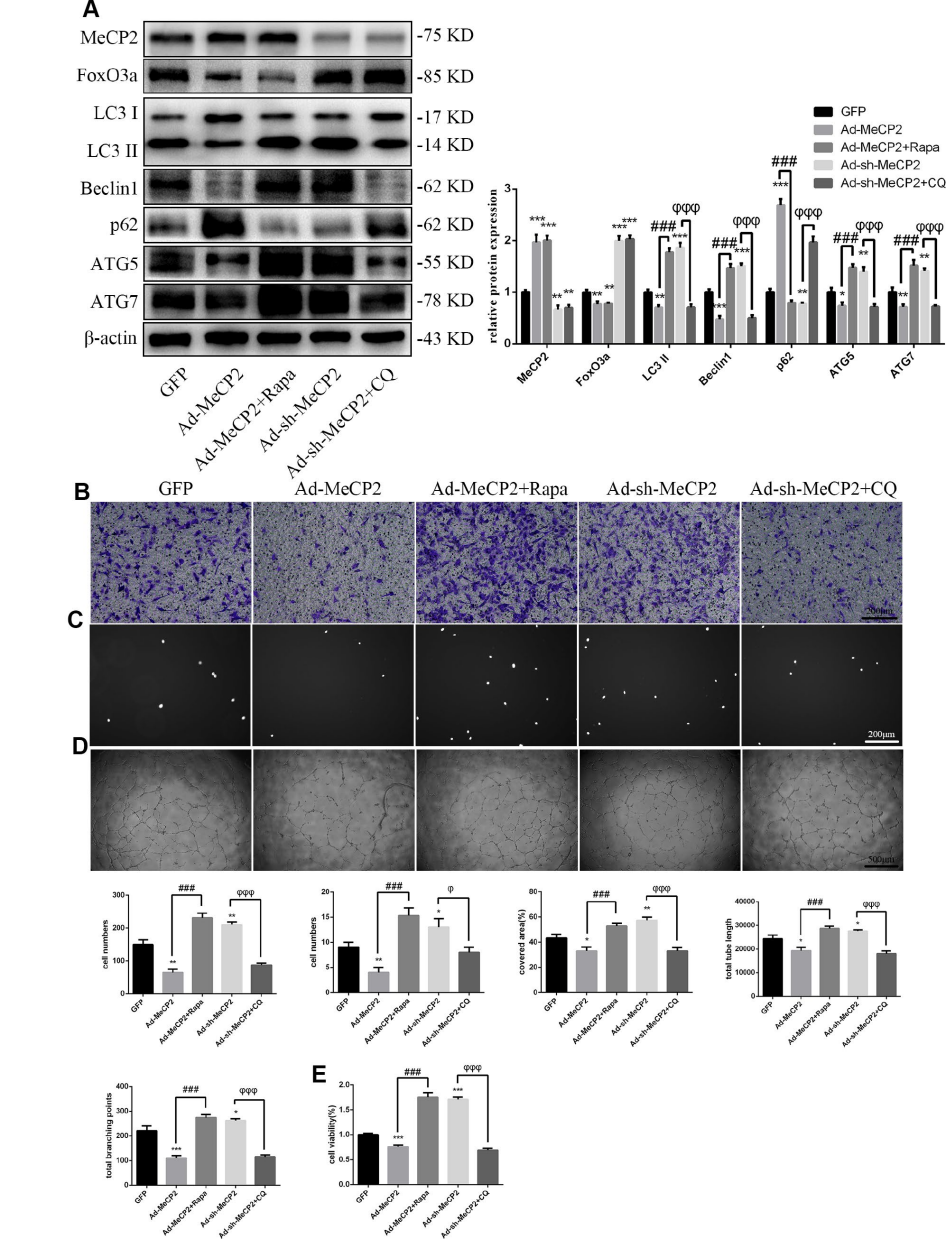
**MeCP2 attenuates EPC functionality through autophagy.** (**A**) FoxO3a, LC3 II, Beclin1, p62, ATG5, and ATG7 protein levels were detected by western blotting after GFP, Ad-MeCP2, Ad-MeCP2 + Rapa, Ad-sh-MeCP2, or Ad-sh-MeCP2 + CQ. (**B**) Cell migration was evaluated by Transwell migration assays after treatment for 48 h. (**C**) Cell adhesion ability was evaluated by matrix adhesion assays after treatment for 48 h. (**D**) Angiogenic ability was evaluated by Matrigel assays after treatment for 48 h. (**E**) Cell viability was evaluated with CCK-8 after treatment for 48 h. *P < 0.05, **P < 0.01, and ***P < 0.001 vs. control. ^#^P < 0.05, ^##^P < 0.01, and ^###^P < 0.001 vs. Ad-MeCP2 treatment. ^φ^P < 0.05, ^φφ^P < 0.01, and ^φφφ^P < 0.001 vs. Ad-sh-MeCP2 treatment.

Similarly, to test the role autophagy plays in the inhibition of EPC function by MeCP2, we used the autophagy activator Rapa or the autophagy inhibitor CQ to block the effects of MeCP2. Our results suggested that autophagy attenuated by Ad-MeCP2 was enhanced after Rapa treatment, and autophagy promoted by Ad-sh-MeCP2 was inhibited after CQ treatment (Figure 6A). Correspondingly, cell functionality was altered, similar to the changes in autophagy flux observed in the group treated with CQ or Rapa (Figure 6B–6E).

### MeCP2 attenuates autophagy and EPC functionality through FoxO3a

**Figure 7:**
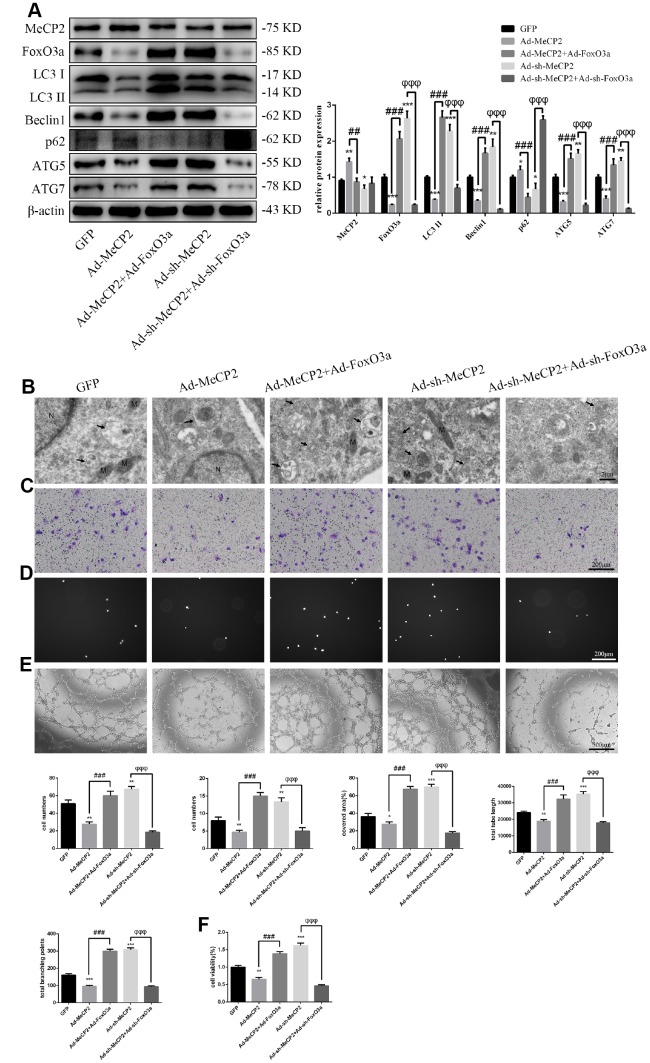
**MeCP2 inhibits autophagy and EPC function through FoxO3a.** (**A**) Protein levels of MeCP2, FoxO3a, LC3 II, Beclin1, p62, ATG5, and ATG7 were detected by western blotting after transfection with Ad-MeCP2 or Ad-sh-MeCP2 or co-transfection with Ad-FoxO3a or Ad-sh-FoxO3a for 48 h. (**B**) EPC ultrastructure was imaged by TEM. The white arrow indicates autophagosomes. N, nucleus; M, mitochondria. (**C**) Cell migration was evaluated by Transwell migration assays after transfection or co-transfection for 48 h. (**D**) Cell adhesion ability was evaluated by matrix adhesion assay after transfection or co-transfection for 48 h. (**E**) Angiogenic ability was evaluated by Matrigel assays after transfection or co-transfection for 48 h. (**F**) Cell viability was evaluated with CCK-8 after transfection or co-transfection for 48 h. *P < 0.05, **P < 0.01, and ***P < 0.001 vs. control. ^#^P < 0.05, ^##^P < 0.01, and ^###^P < 0.001 vs. Ad-MeCP2 treatment. ^φ^P < 0.05, ^φφ^P < 0.01, and ^φφφ^P < 0.001 vs. Ad-sh-MeCP2 treatment.

Our results indicated that MeCP2 attenuates autophagy and EPC functionality. Thus, we next examined the mechanism of autophagy attenuation induced by MeCP2 by employing adenoviral vectors harboring Ad-MeCP2 and Ad-sh-MeCP2 to transfect EPCs. At the same time, adenoviral vectors harboring Ad-FoxO3a and Ad-sh-FoxO3a were used to co-transfect corresponding EPCs. As a result, we found that protein levels associated with autophagy were reversed after co-transfection (Figure 7A); TEM showed that autophagy was attenuated in the group treated with Ad-MeCP2, which was reversed in the group co-transfected with Ad-MeCP2 and Ad-FoxO3a. Moreover, autophagy was elevated in the group treated with Ad-sh-MeCP2 and this tendency was reversed in the group co-transfected with Ad-sh-MeCP2 and Ad-sh-FoxO3a (Figure 7B). Correspondingly, cell migration, adherent ability, angiogenic ability, and cell viability were also affected alongside autophagy (Figure 7C–7F).

### MeCP2 inhibits FoxO3a transcription by promoter hypermethylation and enhancing H3K9 dimethylation in nucleosomes

**Figure 8:**
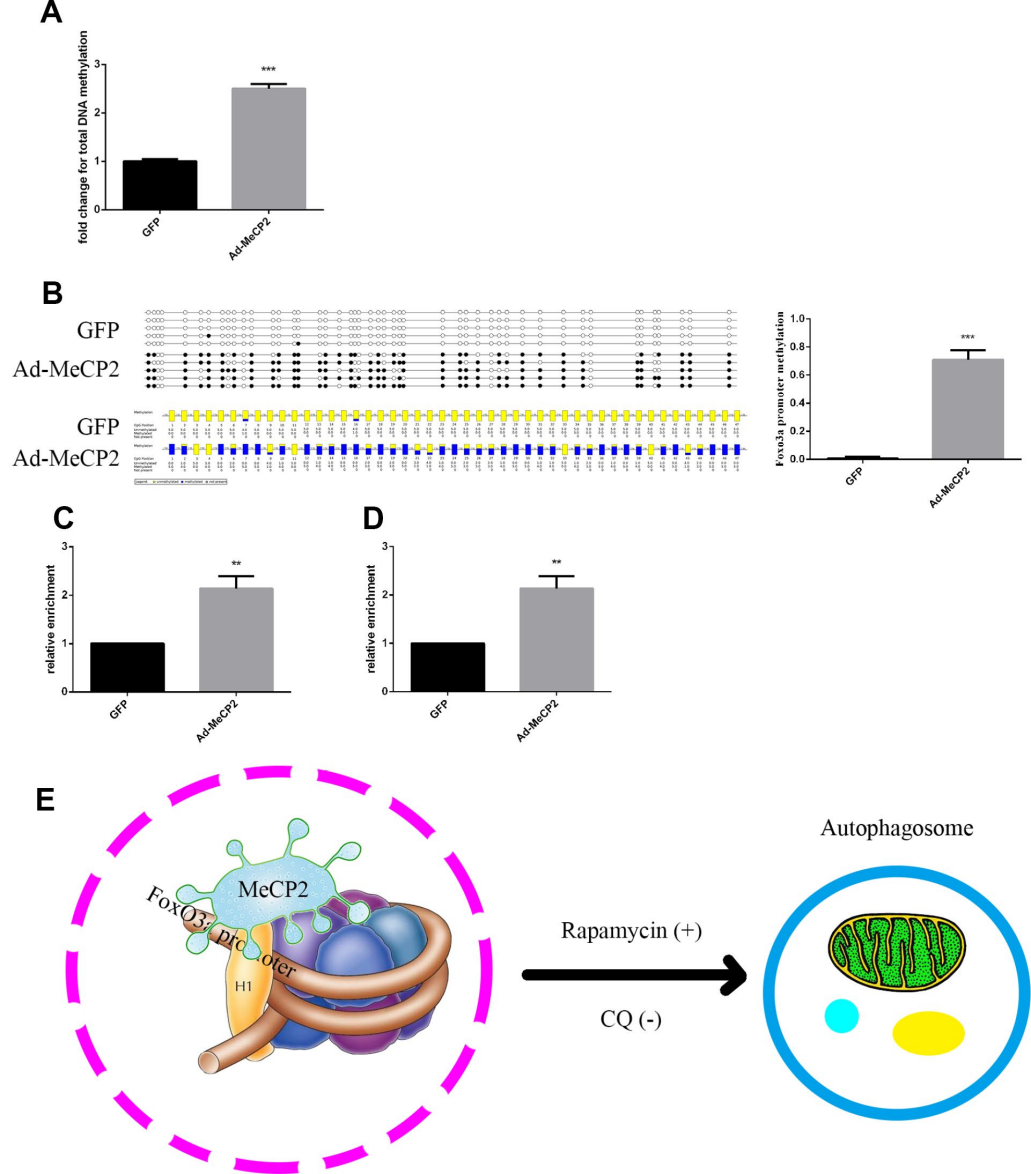
**MeCP2 inhibits FoxO3a transcription by promoting FoxO3a promoter methylation and enhancing H3K9 dimethylation.** (**A**) Genome methylation was detected after MeCP2 overexpression. (**B**) DNA methylation was detected by BSP after MeCP2 overexpression. (**C**) ChIP of MeCP2 binding to the FoxO3a promoter after MeCP2 overexpression. (**D**) ChIP demonstrated H3K9me2 enrichment on the FoxO3a promoter after MeCP2 overexpression. (**E**) Proposed mechanism. *P < 0.05, **P < 0.01, and ***P < 0.001 vs. control.

DNA sequence analysis indicated that there was a CpG island within the FoxO3a promoter region 2000 bp upstream of the transcriptional start site. MeCP2, a transcription factor able to bind CpG islands and enhance methylation, may bind to the CpG island and inhibit gene expression. Our results showed that MeCP2 overexpression in EPCs led to higher DNA methylation compared with that of corresponding GFP-expressing controls (Figure 8A). Moreover, BSP experiments revealed that the methylation status of the FoxO3a promoter was significantly enhanced by MeCP2 overexpression (Figure 8B). We then preformed ChIP to further test MeCP2 binding to the FoxO3a promoter and found that MeCP2 was enriched at the CpG island in the FoxO3a promoter region (Figure 8C). We also preformed ChIP to test the influence of MeCP2 on histone methylation of the FoxO3a promoter and found that MeCP2 enhanced the presence of H3k9me2 in nucleosomes of the FoxO3a promoter (Figure 8D). Our findings suggest that MeCP2 attenuated autophagy by promoting FoxO3a promoter methylation and enhancing H3K9 dimethylation, creating a locally repressive chromatin environment to inhibit FoxO3a transcription (Figure 8E).

## DISCUSSION

Atherosclerosis begins with impaired vascular endothelial function and structure [33, 36, 37]. In recent years, several studies have revealed that EPCs play a crucial role in the replacement of injured vascular endothelial cells [38]. EPCs repair endothelial injury, reduce atherosclerotic plaque, and promote angiogenesis by replacing impaired endothelial cells [39, 40]. Animal experiments have demonstrated that EPCs can repair injured endothelial cells and improve angiogenesis during ischemia [41]. Numerous preclinical and clinical studies have evaluated the therapeutic application of EPCs in the treatment of acute coronary syndrome, chronic myocardial ischemia, cardiomyopathy, and cerebral and peripheral vascular diseases [42, 43]. Further research has shown that the ability of EPCs to replace impaired endothelial cells depends on their number and functionality [44]. However, due to limited EPC capacity, this strategy often leads to insufficient efficacy. Therefore, enhancing EPC function is of great importance in improving the outcomes of EPC transplantation therapy.

It has been previously reported that autophagy also limits atherosclerotic plaque formation by preventing endothelial apoptosis, senescence, and inflammation [5]. On the other hand, defective autophagy promotes apoptosis and accelerated senescence [6]. Our results showed that autophagy promotes EPC cell migration, adherent ability, angiogenic ability, and cell viability, which is consistent with previous studies [45, 46]. In our senescence experiments, we found MeCP2 levels increased but FoxO3a levels decreased. Previous studies have reported that FoxO3a improves cell function by promoting autophagy. For example, ischemic preconditioning can ameliorate renal ischemia/ reperfusion injury by promoting autophagy through the FoxO3a pathway [47]. Therefore, we used Ad-FoxO3a to verify the effects of FoxO3a and confirmed that FoxO3a improves cell function via autophagy in EPCs. Similarly, autophagy activation was found to exhibit a cytoprotective role in premature senescent cells and an increase in autophagy was intimately linked to the activation of the FoxO3a transcription factor [48].

MeCP2 is an important transcriptional factor, as evidenced by Rett syndrome, which is caused by MeCP2 mutations [14]. Previously, MeCP2 was mainly studied with regards to the nervous system. Via our replicative senescence experiments employing the adenoviral vectors Ad-MeCP2 and Ad-sh-MeCP2, we confirmed that MeCP2 overexpression attenuated FoxO3a levels and autophagy, which resulted in decreased EPC functionality. In addition, MeCP2 inhibited angiogenesis in EPCs, which is consistent with the results reported by Volkmann et al. [49]. Our study is the first to expand its function to the endothelial system.

Finally, we used ChIP to verify the inhibitory mechanism of MeCP2 on FoxO3a by utilizing the FoxO3a promoter DNA library and MeCP2 as a methylated CpG binding protein. Our results revealed a feasible mechanism of MeCP2 interaction with FoxO3a for the first time. MeCP2 directly binds the FoxO3a promoter region, increasing promoter methylation and enhancing H3K9 dimethylation to create a repressive chromatin environment that inhibits transcription. Although our results suggest that MeCP2 is directly associated with FoxO3a, there were several limitations to our study. First, the EPCs used in this study are derived from human umbilical cord blood, and therefore senescence in these cells is a function of culture conditions and does not reflect tissue age. ChIP illuminated how MeCP2 overexpression inhibits FoxO3a; however, it remains to be determined how silencing MeCP2 promotes FoxO3a expression. Moreover, MeCP2 levels were reversed slightly when cells were co-transfected with FoxO3a at the same concentration as MeCP2 (Figure 7A). We speculated that FoxO3a possesses MeCP2 feedback regulation but did not verify the hypothesis further. MeCP2 may also interact with other signaling pathways that are involved in autophagy regulation.

Through researching the literature and analyzing senescent EPCs, we were able to shed light on the mechanism underlying MeCP2 and FoxO3a interaction and their relationship with autophagy. We believe that these findings can further expanding the field of autophagy and improve EPC autophagy processes, which may contribute to the development of cellular therapies for AS. Furthermore, the MeCP2 and FoxO3a pathway may also have implications for other cells and diseases.

## CONCLUSIONS

MeCP2 attenuated autophagy by promoting FoxO3a promoter methylation and enhancing H3K9 dimethylation, which inhibited cell functionality of EPCs.

## MATERIALS AND METHODS

### EPC culture and identification

This study was approved by the Ethics Committee of Xinhua Hospital Affiliated with Shanghai Jiao Tong University School of Medicine. EPCs were isolated from human umbilical cord blood by density gradient centrifugation with Histopaque-1077 (Sigma-Aldrich, St. Louis, MO), suspended in complete endothelial cell growth medium-2 (EGM-2; Lonza, Basel, Switzerland), and then seeded in 6-well plates. Cells from approximately 10 mL of cord blood were plated per well. The medium was changed every 3 days and cells were subcultured at a 1:3 ratio. EPC markers were detected by immunofluorescence and flow cytometry. For immunofluorescence assays, antibodies against CD31, CD34, von Willebrand factor (VWF), vascular endothelial growth factor receptor 2 (VEGFR2), and CD133 were purchased from Cell Signaling Technology (Danvers, MA). For flow cytometry, antibodies against CD34, CD133, and VEGFR2 were purchased from Invitrogen (Carlsbad, CA). Dual staining was used to assay the uptake of acetylated low-density lipoprotein (ac-LDL) labeled with the fluorescent probe 1,1′-dioctadecyl-3,3,3′,3′-tetramethyl-indocarbocyanine perchlorate (Dil-ac-LDL; Invitrogen) and the binding of fluorescein isothiocyanate (FITC)-conjugated UEA-1 (Sigma-Aldrich). EPCs were incubated with Dil-ac-LDL (10 μg/mL) for 4 h at 37 °C and then fixed with 4% paraformaldehyde for 20 min. Afterwards, the cells were incubated with FITC-UEA-1 (10 μg/mL) for 1 h and then stained with DAPI for 10 min at room temperature.

### Western blot analysis

Cellular proteins were extracted using immunoprecipitation assay buffer and then approximately 30 μg of protein (per well) was loaded onto 8–12% sodium dodecyl sulfate-polyacrylamide gels for electrophoresis. Proteins were transferred to polyvinylidene fluoride (PVDF) membranes (Millipore, Burlington, MA) and then sequentially detected by primary antibodies, secondary antibodies, and enhanced chemiluminescence substrate (Millipore). Antibodies against MeCP2 (3456), FoxO3a (2497), LC3 (3868), Beclin1 (3495), p62 (8025), ATG5 (12994), ATG7 (8558), and β-actin (3700) as well as anti-mouse and anti-rabbit secondary antibodies were purchased from Cell Signaling Technology. β-actin was used as an internal control.

### Adenovirus transfection

Adenoviral vectors containing green fluorescent protein (Ad-GFP) and harboring wild-type MeCP2 (Ad-MeCP2), MeCP2 short hairpin RNA (Ad-sh-MeCP2), wild-type FoxO3a (Ad-FoxO3a), and FoxO3a short hairpin RNA (Ad-sh-FoxO3a) were purchased from Hanheng Biotechnology (Shanghai, China). Ad-GFP was used as a control. Cells were transfected or co-transfected for 6 h and then incubated with fresh medium. Cellular proteins were extracted 48 h after transfection and then protein expression levels were analyzed by western blotting. The following shRNA sequences were used, MeCP2-shRNA 1, 5′-GCTCTGCT GGGAAGTATGATGTGTA-3′; MeCP2-shRNA 2, 5′-G GAAGCTCCTTGTCAAGATGC-3′; MeCP2-shRNA 3, 5′-GACATTGTTTCATCCTCCATGCCAA-3′; and Ad-MeCP2, NM_004992.3.

### Cell treatment

To study the protective role of autophagy on cells, cells were treated with 1 μM rapamycin (Rapa) + Ad-sh-FoxO3a or Ad-MeCP2 or 10 μM chloroquine (CQ) + Ad-FoxO3a or Ad-sh-MeCP2.

### Cell viability assays

The Cell Counting Kit-8 (CCK-8; Dojindo, Kumamoto, Japan) was used to analyze cell viability after various treatments. Treated cells (1 × 10^4^/well) were suspended in 100 μL fresh medium. After 24 h, CCK-8 (10 μL/well) was added for 3 h of additional incubation and then absorbance was measured at 450 nm.

### Migration assays

Transwell plates (Corning Inc., Corning, NY) were used to observe cell migration. Complete fresh EGM-2 (600 μL) was added to the bottom chambers and treated cells (5 × 10^4^/well) suspended in 200 μL serum free medium were added to the top chamber. After incubating at 37 °C for 12 h, transmigrated cells were fixed in 4% paraformaldehyde (Beyotime, Jiangsu, China) and stained with crystal violet (Beyotime). Three random microscopic fields were selected, and the stained cells were counted.

### Matrigel angiogenesis assays

Matrigel™ (50 μL; BD Biosciences, Franklin Lakes, NJ) was added to the wells of 96-well plates and incubated at 37 °C for 30 min. Then, treated cells (2 × 10^4^/well) were seeded on the Matrigel and incubated at 37 °C. Images were acquired after 8 h.

### Matrix adhesion assay

Adhesion assays were performed to evaluate cell adhesion ability. Equal cell numbers were seeded onto fibronectin-coated 6-well plates. After incubation at 37 °C for 30 min, the cells were gently washed three times with PBS (Gibco; Thermo Fisher Scientific, Waltham, MA) and fixed in 4% paraformaldehyde (Beyotime). Adherent cells were detected by staining with DAPI (Beyotime). Three random microscopic fields were selected, and the stained cells were counted.

### Transmission electron microscopy (TEM)

After treatment, cells were not rinsed with PBS but immediately fixed in electron microscopy stationary liquid (Servicebio, Woburn, MA). Cells were collected into centrifuge tubes with a cell scraper and centrifuged for 5 min, after which the supernatant was discarded. Fresh stationary liquid was added to the precipitate and then samples were fixed for 2 h. Samples were centrifuged, and the cell pellet was embedded in 1% agarose. The samples were washed with PBS and post-fixed with 1% OsO_4_, after which they were washed with PBS and dehydrated with a graded ethanol series (50%, 70%, 80%, 90%, and 95%). The cells were infiltrated with 1:1 acetone:EMBed812 for 2 h, 2:1 acetone:EMBed812 overnight, and pure EMBed812 for 6 h, followed by incubation in a 37 °C oven overnight and baking in a 60 °C oven for 48 h. The samples were then cut into ultrathin sections (60–80 nm) with an ultramicrotome (Leica, Wetzlar, Germany) and stained with uranyl acetate in pure ethanol for 15 min. Next, the samples were rinsed with distilled water, stained with lead citrate for 15 min, rinsed with distilled water again, and then observed with TEM (Hitachi, Tokyo, Japan) after air-drying the sections overnight.

### Genome methylation assay

Genomic DNA was isolated using the Genomic DNA Mini Preparation Kit (Beyotime), after which a genome methylation assay was performed using the MethylFlash Methylated DNA Quantification Kit (Epigentek, Farmingdale, NY) according to manufacturer’s instructions.

### Chromatin immunoprecipitation (ChIP)

The ChIP assay kit was purchased from Thermo Fisher Scientific. Treated cells were fixed with 1% formaldehyde and sheared by sonication. MeCP2 antibody (ab2828; Abcam, Cambridge, UK) was used to immunoprecipitate the MeCP2-DNA complex and H3K9me2 antibody (ab1220; Abcam) was used to immunoprecipitate the histone-DNA complex. Next, the complexes were filtered out from the DNA fragments by protein G agarose beads. MECP2-DNA and histone-DNA complex cross-linking was reversed, and the DNA strands were purified. qPCR was performed to quantify the ChIP products. Primers used for the FoxO3a promoter were as follows, (-699 to -528) forward, 5′-CAAACCTTTTGGTGCCTGAT-3′ and reverse, 5′-GTG TCCGGTTCCCTGTTAGA-3′.

### Bisulfite sequencing PCR (BSP)

After transcription with GFP and Ad-MeCP2, DNA was isolated using the Genomic DNA Mini Preparation Kit with Spin Column (Beyotime, Jiangsu, China) and bisulfite converted with the EZ DNA Methylation-Gold Kit (Zymo Research, Irvine, CA). Primers for a CpG island within 2000 bp upstream of the initiation codon of FoxO3a were designed to amplify the DNA. The primer sequences were as follows, (-1000 to -700) forward, 5′-GTTAGGTTAGGAAAGGGGAG-3′ and reverse, 5′-CCACCTCCCAATCCTACC-3′. PCR products were subcloned into the pCR2.1 vector with a TopoTA Cloning Kit (Invitrogen). Ten colonies were picked, plasmid DNA was purified, and then the DNA was sequenced. The degree of methylated DNA was analyzed using the QUantification tool for Methylation Analysis.

### Statistical analysis

Data are expressed as the mean ± standard error. Comparisons between two groups were performed using the independent samples *t*-test. P values < 0.05 were considered statistically significant. Data were collected from at least three independent experiments.

### Ethics approval

This study was approved by the Ethics Committee of Xinhua Hospital Affiliated with Shanghai Jiao Tong University School of Medicine. Umbilical cord blood was obtained after mothers provided written informed consent.

## Supplementary Material

Supplementary Figures
