# Supplementary material for: MeCP2 inhibits cell functionality through FoxO3a and autophagy in endothelial progenitor cells
Source: Aging (Albany NY). 2019 Sep 2;11(17):6714–33. doi: 10.18632/aging.102183 (PMC6756911; doi:10.18632/aging.102183)
Supplement: Supplementary Figures [file aging-11-102183-s001.pdf]

## SUPPLEMENTARY FIGURES

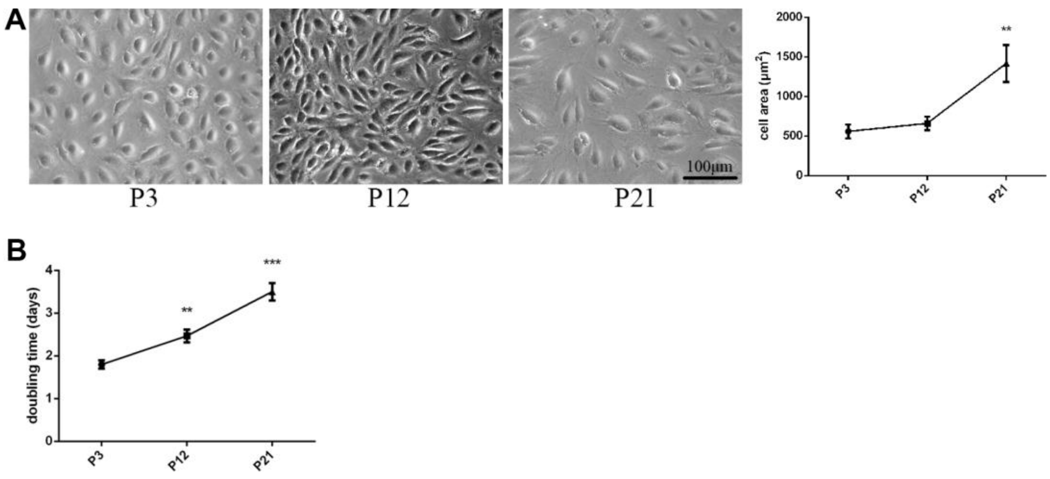


**Supplementary Figure 1. Changes in size and proliferation rates after passaging.** (**A**) Changes in size of aging EPCs. (**B**) Changes in proliferation rates of aging EPCs.

F2

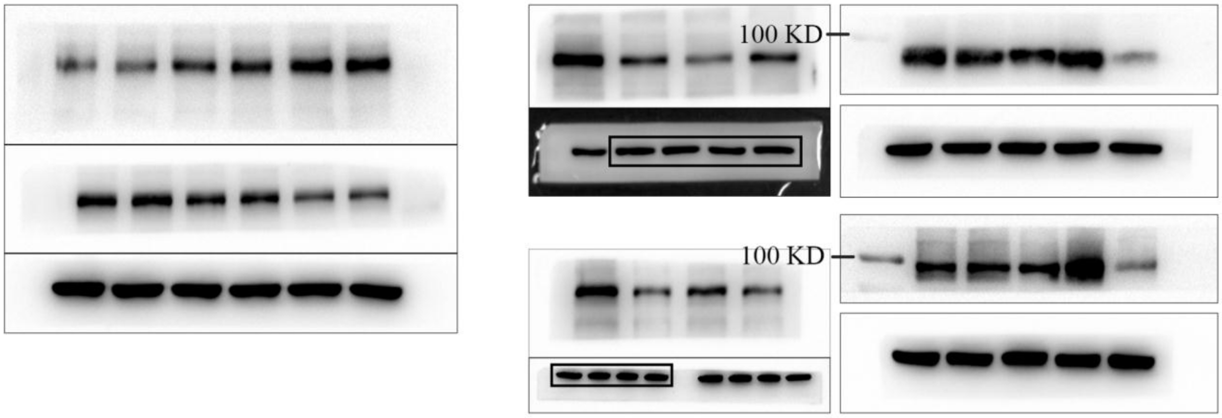


F3

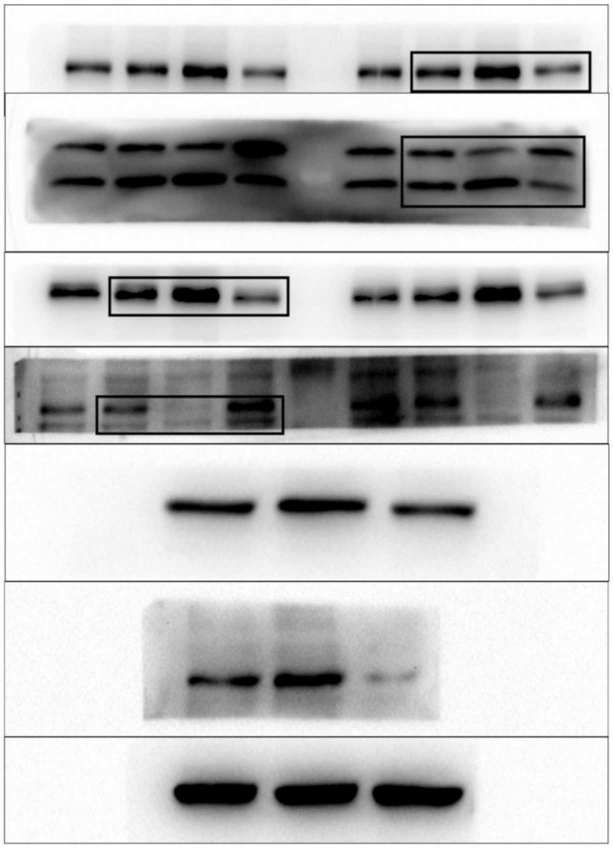

F4

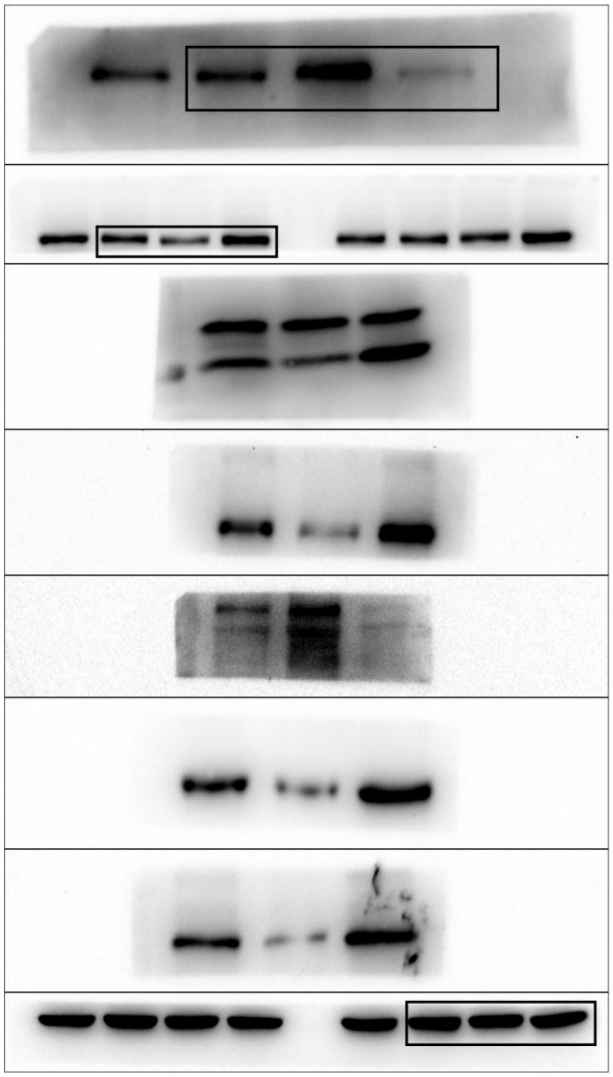

**Supplementary Figure 2. Full western blot images.**

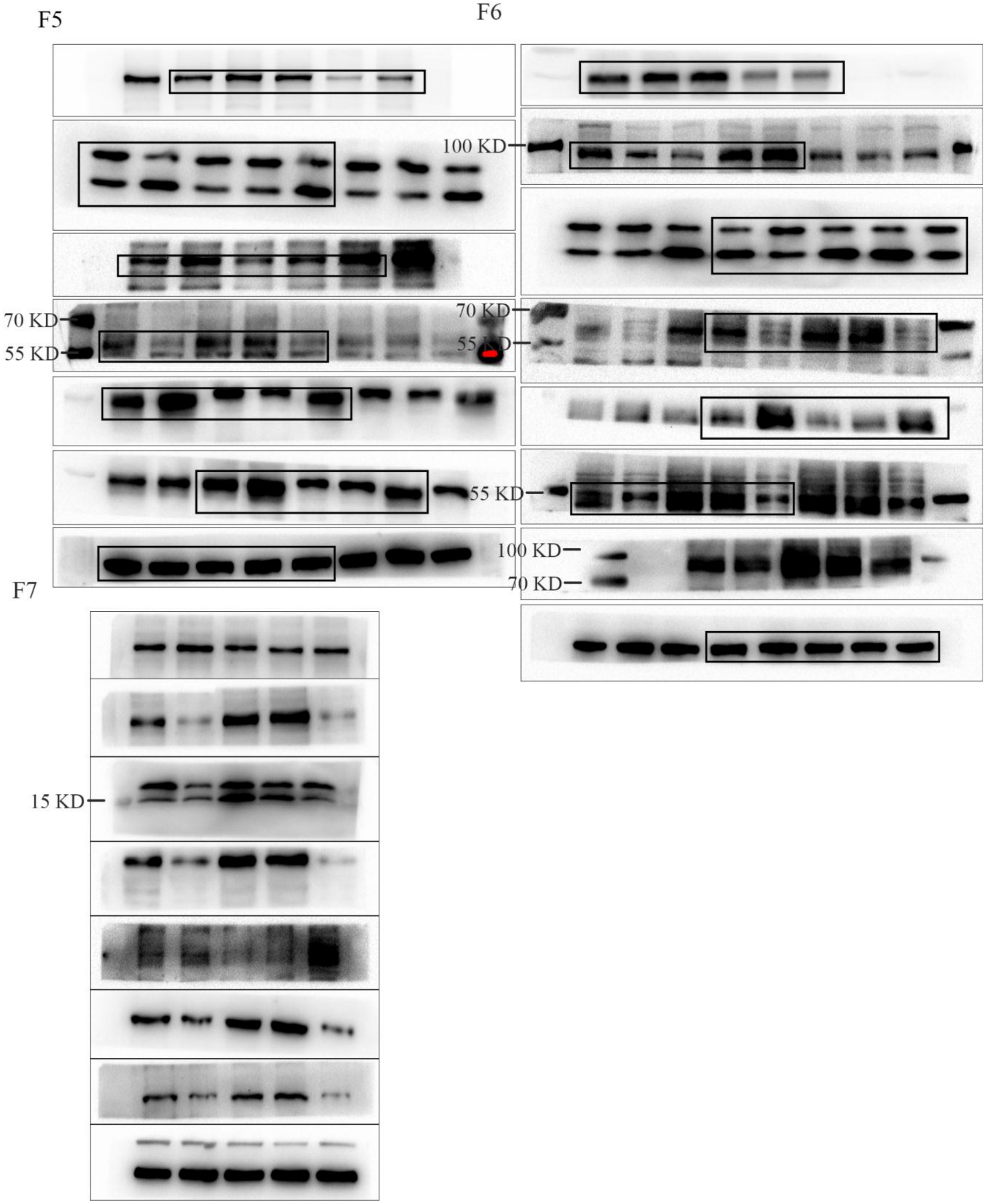


**Supplementary Figure 3. Full western blot images.**
